# MiR-21-5p Links Epithelial-Mesenchymal Transition Phenotype with Stem-Like Cell Signatures via AKT Signaling in Keloid Keratinocytes

**DOI:** 10.1038/srep28281

**Published:** 2016-09-06

**Authors:** Li Yan, Rui Cao, YuanBo Liu, LianZhao Wang, Bo Pan, XiaoYan Lv, Hu Jiao, Qiang Zhuang, XueJian Sun, Ran Xiao

**Affiliations:** 1 Research Center of Plastic Surgery Hospital, Chinese Academy of Medical Sciences & Peking Union Medical College, Beijing, P.R. China; 2 Extremities Plastic and Reconstructive Center, Plastic Surgery Hospital, Chinese Academy of Medical Sciences & Peking Union Medical College, Beijing, P.R. China; 3 Comprehensive Treatment Center of Scar, Plastic Surgery Hospital, Chinese Academy of Medical Sciences & Peking Union Medical College, Beijing, P.R. China; 4 Auricular Plastic and Reconstructive Surgery Center, Plastic Surgery Hospital, Chinese Academy of Medical Sciences & Peking Union Medical College, Beijing, P.R. China

## Abstract

Keloid is the abnormal wound healing puzzled by the aggressive growth and high recurrence rate due to its unrevealed key pathogenic mechanism. MicroRNAs contribute to a series of biological processes including epithelial-mesenchymal transition (EMT) and cells stemness involved in fibrotic disease. Here, using microRNAs microarray analysis we found mir-21-5p was significantly up-regulated in keloid epidermis. To investigate the role of miR-21-5p in keloid pathogenesis, we transfected miR-21-5p mimic or inhibitor in keloid keratinocytes and examined the abilities of cell proliferation, apoptosis, migration and invasion, the expressions of EMT-related markers vimentin and E-cadherin and stem-like cells-associated markers CD44 and ALDH1, and the involvement of PTEN and the signaling of AKT and ERK. Our results demonstrated that up-regulation or knockdown of miR-21-5p significantly increased or decreased the migration, invasion and sphere-forming abilities of keloid keratinocytes, and the phenotype of EMT and cells stemness were enhanced or reduced as well. Furthermore, PTEN and p-AKT were shown to participate in the regulation of miR-21-5p on EMT phenotypes and stemness signatures of keloid keratinocytes, which might account for the invasion and recurrence of keloids. This molecular mechanism of miR-21-5p on keloid keratinocytes linked EMT with cells stemness and implicated novel therapeutic targets for keloids.

Keloids are the results of abnormal wound healing processes, which keep growing like tumors beyond the boundaries of the original wound and can cause severe pain and pruritus. The key pathogenic mechanism of the invasion and recurrence of keloids is still unknown, and there is no satisfactory treatment for keloids^1^,^2^. The epithelial-mesenchymal transition (EMT) is a biological process, in which epithelial cells disintegrate their cell-cell adhesion, losing the epithelial markers (e.g., E-cadherin) and gaining the mesenchymal markers (e.g., vimentin) with up-regulated EMT- associated regulative snail family transcripts snail1 and snail2, then the cytoskeleton has been remodeled and the cells obtain a stronger ability to migrate^3^. EMT is critical for tumor metastasis and fibrosis^4^,^5^, and occurs during skin wound healing^6^. EMT-derived cells exhibit similar functions to mesenchymal stem cells (MSCs) with multi-lineage differentiation potential and the ability to migrate towards tumor cells and wound sites^7^. The up-regulation of EMT-related genes and an increased motility have been reported in keloid keratinocytes^8^. Our previous study provided the evidence of the presence of EMT in keloid epidermis and skin appendages, suggesting that EMT might be involved in keloid formation and epithelial cells from epidermis and skin appendages undergoing EMT could be the sources for a fraction of the fibroblasts/myofibroblasts with the invasive property^9^. However, the underlying mechanisms involving EMT in the pathogenesis of keloids need to be elucidated.

MicroRNAs (miRNAs) are small non-coding RNAs about 21–24 nucleotides (nt) in length, which regulate gene expression at the post-transcriptional and translational level, and associate with many biological functions. Recent studies demonstrated that some of the miRNAs could play oncogenic roles in cancers by regulating cell growth, EMT, and cancer stem cells (CSCs) related to cancer progression and metastasis^10^,^11^. CSCs are found in various cancers with the characteristics of stem cells including the abilities of self-renewing and producing differentiated progeny, and the induction of EMT has been proven to be associated with the generation of stem-like cells^12^,^13^. Therefore, miRNA pathways are most likely to be involved in regulating EMT-associated stem cells, which direct us to analyze differential expression profile of microRNA in keloid epidermis. MicroRNA-21 (miR-21) is one of the oncogenic miRNAs overexpressed in most types of human cancers and it could be a potential molecular prognostic marker and a target for cancer therapy^14^,^15^. MiR-21 has been found to play crucial roles by regulating the target proteins in various signaling pathways. The phosphatase and tensin homolog (PTEN), a negative regulator of EMT and protein kinase B (Akt) signaling pathways, is one of the target genes of miR-21^16^. MiR-21 is also overexpressed in fibrotic diseases, including kidney, pulmonary, cardiovascular, liver, and skin fibrosis^17^,^18^,^19^,^20^,^21^, as well as in keloid fibroblasts whose proliferation and apoptosis were affected by miR-21 through regulating PTEN^22^. Nevertheless, there is no report on the expression and regulative function of miR-21 in keloid epithelial cells.

In the current study, we aimed to investigate the differential expression of microRNAs in the keloid epidermis compared with that in the normal skin epidermis. For the selected, significantly expressed microRNA-21-5p (miR-21-5p), the effects on the proliferation, apoptosis and ability of migration and invasion of keloid keratinocytes and normal skins keratinocytes were evaluated. The examinations were carried out to confirm the roles of miR-21-5p in the regulation for the phenotypes of EMT and the stem-like cells in these keratinocytes. And the involvement of miR-21-5p in the regulation of PTEN, the signaling of AKT and extracellular signal regulated kinase (ERK) in keloid keratinocytes were further investigated.

## Results

### Differential expression profile of microRNA in keloid epidermis

**Figure 1:**
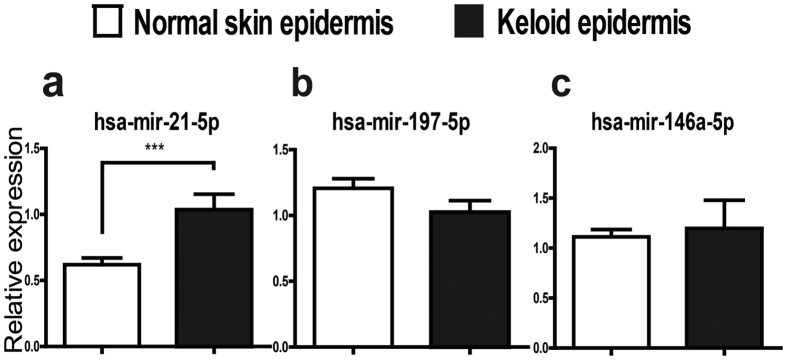
Differential expression of microRNAs in the epidermis derived from keloids and normal skins. (**a–c**) 3 up-regulated miRNAs were detected in epidermis from 8 keloids and 8 normal skins by SYBR Green quantitative RT-PCR (qRT-PCR). The expression of each miRNA was normalized by the expression of U6 miRNA. Statistical significance was obtained using Independent-Samples t-test. Data was shown as the mean ± SEM of 8 individual samples from 3 replicates. (***p < 0.001).

The miRNA microarray detected 8 overexpressed miRNAs (*P* < 0.05 and fold change >2) in keloid epidermis compared with those in normal skin epidermis (Table 1). The expression of mir-21-5p was verified to be significantly higher in keloid epidermis than in normal skin epidermis (Fig. 1a, *P* = 0.0006) by quantitative reverse transcription-polymerase chain reaction (qRT-PCR) analysis. We also detected the expressions of mir-197-5p and mir-146a-5p, and found there was no significant difference between keloid epidermis and normal skin epidermis (Fig. 1b,c, *P* = 0.0827 and *P* = 0.6394).

### Influences of miR-21-5p mimic on proliferation, apoptosis, and the migration and invasion ability of keratinocytes from keloid and normal skin

**Figure 2:**
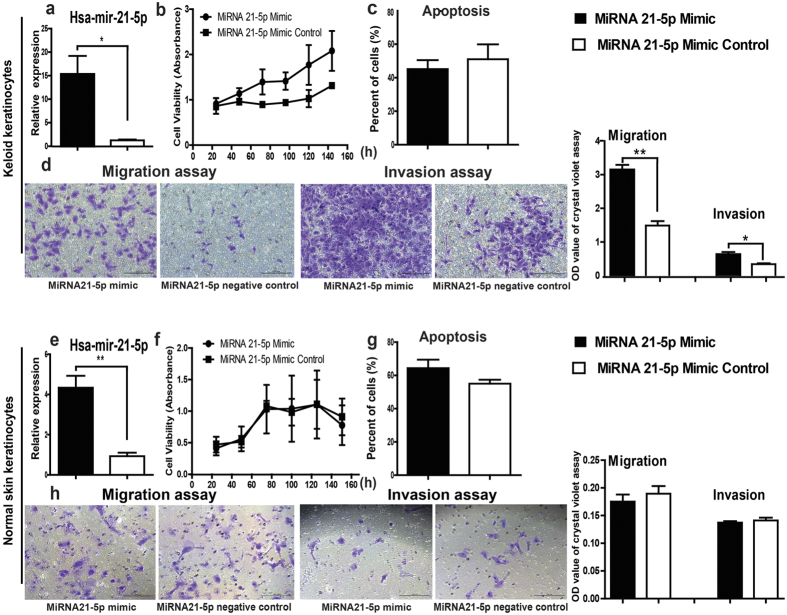
The effects of miR-21-5p mimic on the proliferation, apoptosis, and the ability of migration and invasion of keratinocytes from keloid and normal skin. After keloid keratinocytes and normal skin keratinocytes were transfected with miR-21-5p mimic (2.5 μl, 25 pmole) and their corresponding negative controls for 72 hours, (**a,e**) the expression of miR-21-5p was detected using TaqManMiRNA Assay System, (**b,f**) the MTS assay and (**c,g**) annexin VPE apoptosis detection were performed. (**d,h**) The migrated and invaded cell numbers were examined using OD595 nm values and the microscope. Statistical significance was obtained using Independent-Samples t-test. Data was shown as the mean ± SEM of 3 independent cells from 3 replicates. Scale bar: 100 μm. (*p < 0.05, **p < 0.01).

MiR-21-5p mimic as well as the corresponding negative control was transfected into keloid keratinocytes and normal skin keratinocytes. Compared with the corresponding negative control, after transfected with miR-21-5p mimic the amount of miR-21-5p increased significantly in keloid keratinocytes (Fig. 2a, *P* = 0.0216) and normal skin keratinocytes (Fig. 2e, *P* = 0.0053). 3-(4, 5-dimethylthiazol-2-yl)-5-(3-carboxymethoxyphenyl)-2-(4-sul-fophenyl) -2H-tetrazolium, inner salt (MTS) assay (Fig. 2b,f) and Annexin V apoptosis detection (Fig. 2c,g) demonstrated that the proliferation and apoptosis of miR-21-5p mimic transfected keratinocytes in both keloid and normal skin groups did not show significant changes compared with the cells transfected with the negative controls (*P* > 0.05). However, the microscopic observation and the OD_595 nm_ (optical density) values showed that the numbers of the migrated and invaded cells in miR-21-5p mimic transfected keloid keratinocytes were significantly higher than those in the negative control transfected ones (Fig. 2d, *P* = 0.0011 and *P* = 0.0167, respectively), but there was no significant changes in miR-21-5p mimic transfected normal skin keratinocytes (Fig. 2h, *P* = 0.49 and *P* = 0.5238, respectively). These findings confirmed that up-regulation of miR-21-5p could enhance the migration and invasion ability of keloid keratinocytes, but could not affect the ability of migration and invasion of normal skin keratinocytes.

### Influences of miR-21-5p inhibitor on proliferation, apoptosis, and the migration and invasion ability of keratinocytes from keloid and normal skin

**Figure 3:**
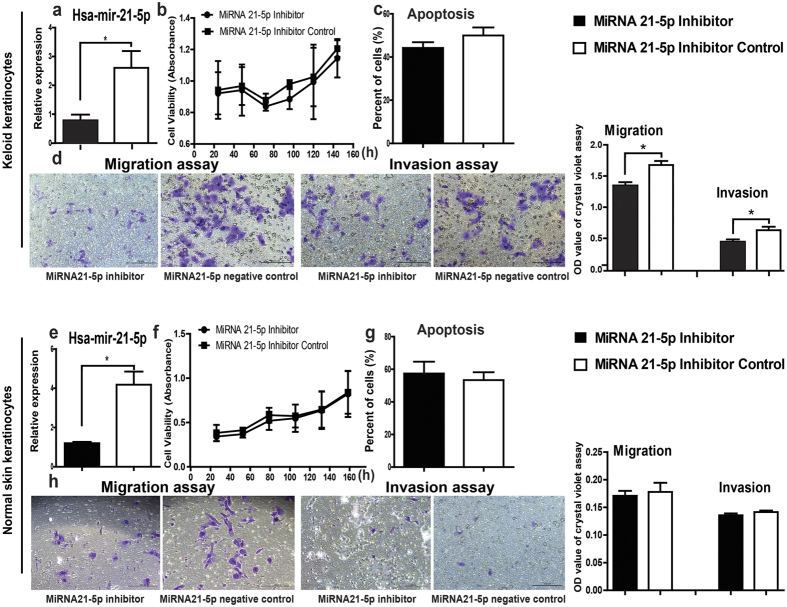
The effects of miR-21-5p inhibitor on the proliferation, apoptosis, and the ability of migration and invasion of keratinocytes from keloid and normal skin. After keloid keratinocytes and normal skin keratinocytes were transfected with miR-21-5p inhibitor (7.5 μl, 75 pmole) and their corresponding negative controls for 48 hours, (**a,e**) the expression of miR-21-5p was detected using TaqManMiRNA Assay System, (**b,f**) the MTS assay and (**c,g**) annexin VPE apoptosis detection were performed. (**d,h**) The migrated and invaded cell numbers were examined using OD595 nm values and the microscope. Statistical significance was obtained using Independent-Samples t-test. Data was shown as the mean ± SEM of 3 independent cells from 3 replicates. Scale bar: 100 μm. (*p < 0.05).

Similarly, miR-21-5p inhibitor as well as the corresponding negative control was transfected into keloid keratinocytes and normal skin keratinocytes. Compared with the corresponding negative control, after transfected with miR-21-5p inhibitor the expression of miR-21-5p decreased significantly in keloid keratinocytes (Fig. 3a, *P* = 0.0419) and normal skin keratinocytes (Fig. 3e, *P* = 0.0116). MTS assay (Fig. 3b,f) and Annexin V apoptosis detection (Fig. 3c,g) demonstrated that the proliferation and apoptosis of the miR-21-5p inhibitor transfected keratinocytes in both keloid and normal skin groups did not show significant changes compared with the cells transfected with the negative controls (*P* > 0.05). However, the microscopic observation and the OD_595 nm_ values showed that the numbers of the migrated and invaded cells in miR-21-5p inhibitor transfected keloid keratinocytes were significantly lower than those in the negative control transfected ones (Fig. 3d, *P* = 0.0172 and *P* = 0.048, respectively), and did not show significant changes in miR-21-5p inhibitor transfected normal skin keratinocytes (Fig. 3h, *P* = 0.7282 and *P* = 0.2574, respectively). These findings confirmed that down-regulation of miR-21-5p could reduce the migration and invasion ability of keloid keratinocytes, but could not affect the ability of migration and invasion of normal skin keratinocytes.

### Regulation of miR-21-5p on EMT phenotype of keratinocytes from keloid and normal skin

**Figure 4:**
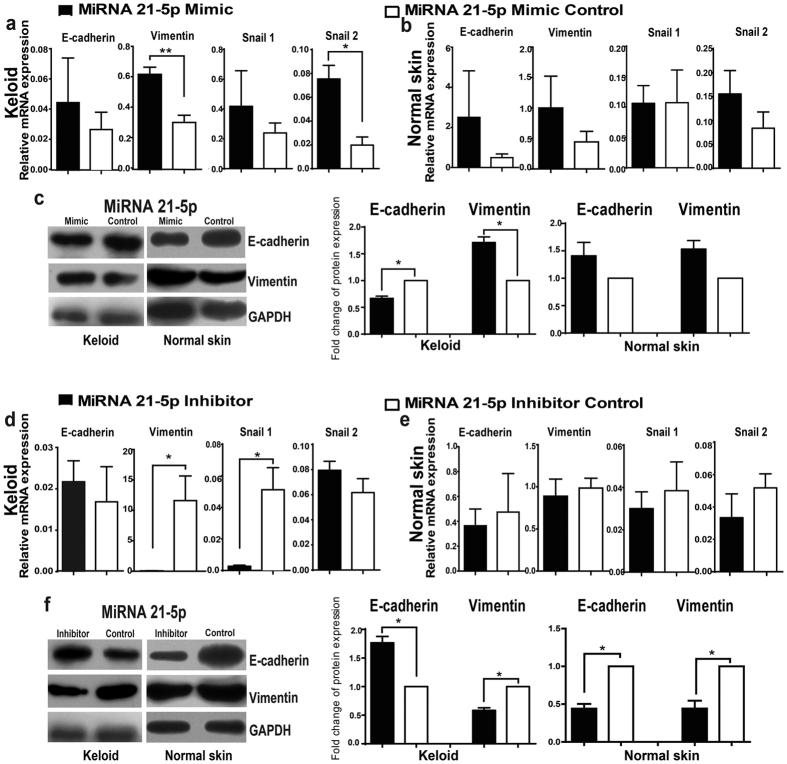
The effect of miR-21-5p on the EMT phenotype of keratinocytes from keloid and normal skin. After keloid keratinocytes and normal skin keratinocytes were transfected with miR-21-5p mimic or inhibitor and their corresponding negative controls for 72 or 48 hours, respectively, the expressions of EMT-related markers E-cadherin, Vimentin, Snail1 and Snail2 in keloid keratinocytes and normal skin keratinocytes were examined by qRT-PCR (**a,b,d,e**) and western blot analysis (**c,f** ), and were normalized by the expression of GAPDH. The western blot data are expressed as fold change relative to the mean value of control group. Statistical significance was obtained using Independent-Samples t-test for qRT-PCR and Paired t-test for western blot analysis. Data was shown as the mean ± SEM of 3 independent cells from 3 replicates. (*p < 0.05, **p < 0.01).

The expressions of EMT-related markers were examined in both the miR-21-5p mimic and inhibitor transfected keratinocytes from keloid and normal skin. qRT-PCR analysis showed that the mRNA of *vimentin* and *snail2* increased significantly in miR-21-5p mimic transfected keloid keratinocytes compared with that in the negative control (Fig. 4a, *P* = 0.0096 and *P* = 0.0138), while the expressions of *E-cadherin* and *snail*1 showed no significant difference between the two groups (Fig. 4a, *P* = 0.6078 and *P* = 0.0765). Results of western blot showed an enhanced expression of vimentin and a decreased expression of E-cadherin in miR-21-5p mimic transfected keloid keratinocytes compared with the negative control (Fig. 4c, *P* = 0.0151 and *P* = 0.0206, respectively). Although there was an increased trend of the expressions of vimentin and E-cadherin in mimic transfected normal skin keratinocytes, both qRT-PCR analysis and western blot did not show significant changes compared with the cells transfected with the negative controls (Fig. 4b, *P* = 0.3688 and *P* = 0.4363; Fig. 4c, *P* = 0.0741 and *P* = 0.2352).

On the contrary, the mRNA of *vimentin* and *snail*1 decreased significantly in keloid keratinocytes transfected with miR-21-5p inhibitor compared with the negative control (Fig. 4d, *P* = 0.0219 and *P* = 0.0257), and there was no significant change in normal skin keratinocytes (Fig. 4e, *P* = 0.7198 and *P* = 0.6191).The expression of *E-cadherin* and *snail*2 at mRNA level showed no significant difference both in keloid keratinocytes (Fig. 4d, *P* = 0.652 and *P* = 0.251) and normal skin keratinocytes (Fig. 4e, *P* = 0.7758 and *P* = 0.3407). However, western blot results showed in miR-21-5p inhibitor transfected keloid keratinocytes the expression of E-cadherin increased (Fig. 4f, *P* = 0.0202) and the expression of vimentin decreased (Fig. 4f, *P* = 0.0116), meanwhile, both the expressions of E-cadherin and vimentin decreased in miR-21-5p inhibitor transfected normal skin keratinocytes (Fig. 4f, *P* = 0.0121 and *P* = 0.0328, respectively). The results suggested that the up- or down-regulation of miR-21-5p could induce or reverse the EMT phenotype of keloid keratinocytes, but not exert similar effect on normal skin keratinocytes.

### Regulation of miR-21-5p on stem-like cell signatures of keratinocytes from keloid and normal skin

**Figure 5:**
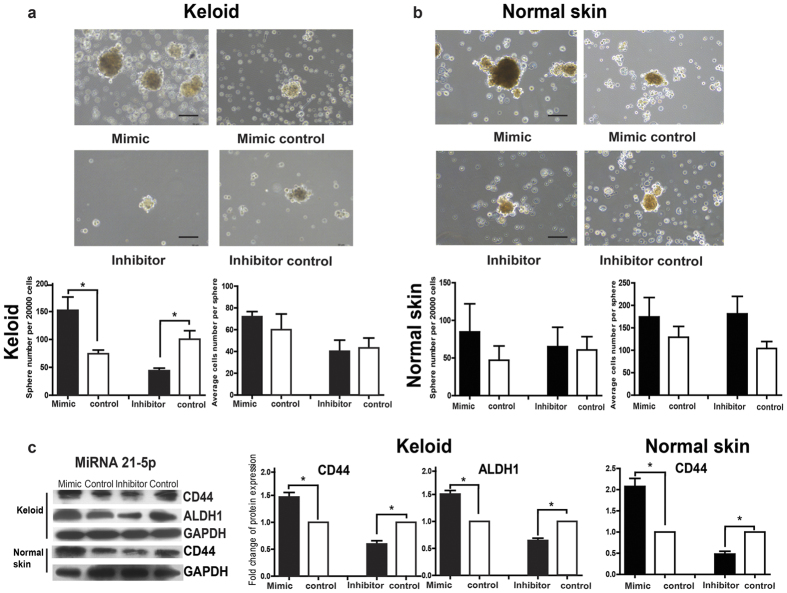
The regulation of miR-21-5p on the sphere formation ability and the stem-like cells phenotype of keratinocytes from keloid and normal skin. After keloid keratinocytes and normal skin keratinocytes were transfected with miR-21-5p mimic or inhibitor and their corresponding negative controls for 72 or 48 hours, respectively, (**a,b**) the average sphere numbers and cells number were detected by the sphere-forming assay. (**c**) The expressions of stem-like cells-associated markers (CD44 and ALDH1) were examined by western blot analysis and normalized by the expression of GAPDH. The western blot data are expressed as fold change relative to the mean value of control group. Statistical significance was obtained using Independent-Samples t-test for sphere-forming assay and Paired t-test for western blot analysis. Data was shown as the mean ± SEM of 3 independent cells from 3 replicates. Scale bar: 100 μm. (*p < 0.05).

The sphere-forming assay, which can be employed to assess clonogenicity, long-term renewal capacities, and multilineage differentiation of cells, was used to evaluate the effects of miR-21-5p on the stem-like cell signatures of keloid keratinocytes and normal skin keratinocytes. Results showed that the average numbers of spheres increased in the miR-21-5p mimic transfected keloid keratinocytes and decreased in the inhibitor transfected keloid keratinocytes (Fig. 5a, *P* = 0.0342 and *P* = 0.0245, respectively). As to the cells of each sphere in keloid keratinocytes, the average number was 72 ± 4.726 in the mimic group and 58 ± 16.46 in its negative control, and 40 ± 10.14 in the inhibitor group and 43 ± 9.062 in its negative control (Fig. 5a). However, the average numbers of spheres had no significant difference in normal skin keratinocytes transfected with the miR-21-5p mimic or inhibitor compared with the negative control (Fig. 5b, *P* > 0.05), and the average cells number of each sphere was 174±43.49 in the mimic and 129±24.17 in its negative control, and 181±38.91 in the inhibitor and 104±15.5 in its negative control (Fig. 5b).

Additionally, compared with the corresponding negative control, the expressions of stem-like keratinocytes-associated markers CD44 and aldehyde dehydrogenase 1 (ALDH1) increased significantly in miR-21-5p mimic transfected keloid keratinocytes (Fig. 5c, *P* = 0.0311 and *P* = 0.0175) and decreased in miR-21-5p inhibitor transfected keloid keratinocytes (Fig. 5c, *P* = 0.0226 and *P* = 0.014, respectively). Although similar expression changes of CD44 were found in miR-21-5p mimic transfected normal skin keratinocytes (Fig. 5c, *P* = 0.0292) and miR-21-5p inhibitor transfected normal skin keratinocytes (Fig. 5c, *P* = 0.0161), the expression of ALDH1 could not be detected in normal skin keratinocytes. The results suggested that the up- or down-regulation of miR-21-5p could enhance or weaken the stem-like cell signatures of keloid keratinocytes, but only playing partial effect on the expression of the stemness marker in normal skin keratinocytes.

### Regulation of miR-21-5p on the EMT and stem-like cells phenotype and the PTEN pathway in keloid keratinocytes

**Figure 6:**
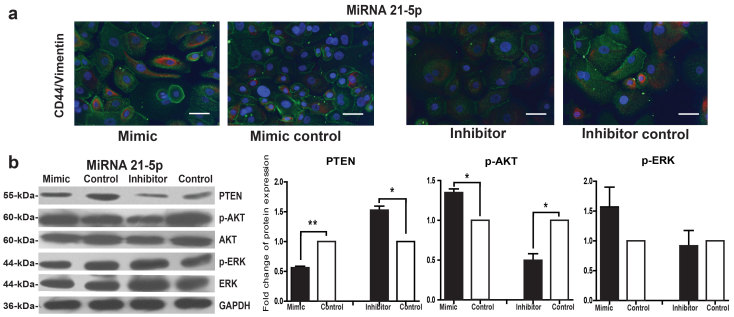
The regulation of miR-21-5p on the EMT and stem-like cells phenotype, PTEN, AKT and ERK of keloid keratinocytes. After keloid keratinocytes were transfected with miR-21-5p mimic and negative mimic control for 72 hours or miR-21-5p inhibitor and the negative inhibitor control for 48 hours, (**a**) immunofluorescence dual staining was used to detect the expression of CD44 (positive green staining) and vimentin (positive red staining) in keloid keratinocytes. Nuclei were visualized by staining with DAPI (blue). Scale bars: 50 μm. (**b**) The expression of PTEN, p-AKT and p-ERK were tested by western blot analysis, and were normalized by the expression of GAPDH and presented as fold change relative to the mean value of control group. Statistical significance was obtained using Paired t-test for western blot analysis. Data was shown as the mean ± SEM of 3 independent cells from 3 replicates. (*p < 0.05, **p < 0.01).

As miR-21-5p mimic induced the EMT and stem-like cells phenotype in keloid keratinocytes, to figure out whether keloid keratinocytes with the EMT phenotype also possess stem-like cell signature, double fluorescence staining for CD44 (green) and vimentin (red) was performed. The results showed the stronger positive staining in miR-21-5p mimic transfected keloid keratinocytes compared with mimic control transfection, and some fibroblast-like cells appeared positive for both CD44 and vimentin (Fig. 6a). There was very weak staining in miR-21-5p inhibitor transfected keloid keratinocytes.

Compared with the corresponding negative control, the expression of PTEN significantly decreased in keloid keratinocytes transfected with miR-21-5p mimic (Fig. 6b, *P* = 0.0033), and increased in the inhibitor transfected keloid keratinocytes (Fig. 6b, *P* = 0.0162), while the expression of phosphorylated AKT (p-AKT) increased in the mimic (*P* = 0.0180), and decreased in the inhibitor transfected keloid keratinocytes (*P* = 0.026). The expression of phosphorylated ERK (p-ERK) showed no significant difference in both mimic and inhibitor transfection cells.

### MiR-21-5p regulates the EMT and stem-like cells phenotype of keloid keratinocytes via the AKT signaling by targeting PTEN

**Figure 7:**
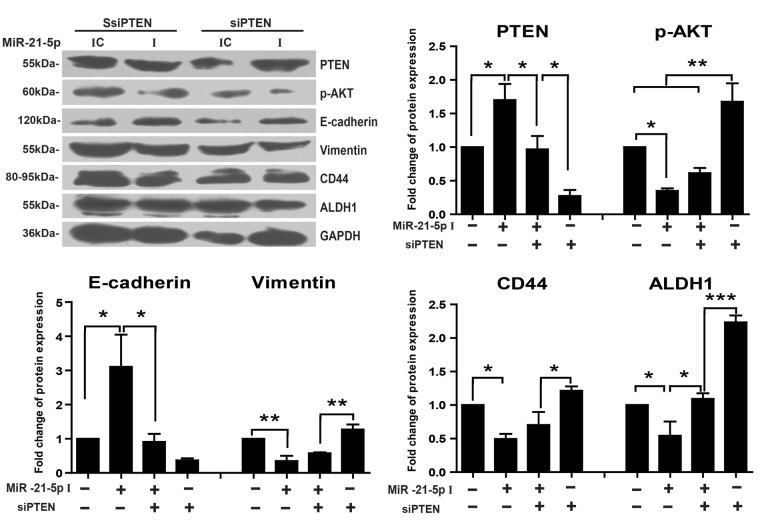
MiR-21-5p regulates the EMT and stem-like cells phenotype of keloid keratinocytes via the AKT signaling by targeting PTEN. The protein expressions of PTEN, p-AKT, EMT-related and stem-like cells associated markers were tested by western blot analysis after keloid keratinocytes were transfected with selected RNA duplex specific for the sequence of PTEN mRNA (siPTEN, 8 μl) or the scramble control siRNA sequence (SsiPTEN) for 24 hours firstly, then transfected with miR-21-5p inhibitor (I) (7.5 μl, 75 pmole) or the negative inhibitor control (IC) for 48 hours. The expressions of protein were normalized by the expression of GAPDH and presented as fold change relative to the mean value of control group transfected with SsiPTEN and IC. Statistical significance was obtained by One-Way ANOVA analysis. Data was shown as the mean ± SEM of 3 independent cells from 3 replicates. (*p < 0.05, **p < 0.01, ***p < 0.001).

The expressions of PTEN, p-AKT, EMT-related and stem-like cell markers were examined after keloid keratinocytes were transfected with miR-21-5p inhibitor and small interfering RNA PTEN (siPTEN) (Fig. 7). The expression of PTEN was significantly increased in cells transfected with miR-21-5p inhibitor and decreased in cells transfected with siPTEN (P < 0.05). Clearly, in the group of keloid keratinocytes transfected with miR-21-5p inhibitor, the expression of p-AKT was significantly decreased (P < 0.05) with the reversed EMT phenotype including enhanced E-cadherin (P < 0.05) and reduced vimentin (P < 0.01), as well as the decreased expressions of stemness marker CD44, and ALDH1 (P < 0.05). Furthermore, when PTEN expression was kept to the control level in the transfection group with both inhibitor and siPTEN, the expressions of E-cadherin and ALDH1 were completely recovered (P < 0.05), whereas p-AKT, vimentin and CD44 got partially recovered. Besides, in the PTEN knockdown only group, there was a remarkable increasing for the expressions of p-AKT (P < 0.01), vimentin (P < 0.01) and ALDH1 (P < 0.001) in keloid keratinocytes.

## Discussion

The challenge in clinical therapy for keloid is its invasive growth and high recurrence rate. It has been found that cancer cells undergoing EMT could invade the surrounding normal tissues and even acquire the CSC property and become more resistant to radiotherapy and chemotherapy; therefore, EMT is considered to be involved in the invasion, recurrence, and drug resistance of cancers^23^. MiR-21 has been reported to regulate a series of biological processes, including cell proliferation, migration, invasion, and CSCs maintenance^14^,^15^. MiR-21 is overexpressed in cancers and significantly associated with advanced clinical stage, lymph node metastasis, and poor patient survival^14^, and is also considered as a novel biomarker and therapeutic target in fibrotic diseases including keloid^22^. In keloid, the underlying mechanisms that epithelial cells undergo EMT and whether these cells gain a stem-like cell phenotype need to be explored. In the current study, we demonstrated that miR-21-5p was overexpressed in keloid epidermis, and in keloid keratinocytes the expressions of markers related to EMT and stem-like cells could be enhanced or inhibited by the mimic or inhibitor of mir-21-5p, which phenotypes were accompanied by the increasing or decreasing of migration and invasion abilities of keloid keratinocytes. These findings suggested that miR-21-5p might contribute to the invasive and recurrence properties of keloids by linking EMT phenotype with stem-like cell signatures in keloid keratinocytes.

Evidence shows that the acquisition and maintenance of stemness in cancer cells profit from EMT^24^. EMT induction in human breast cancer cells could enhance drug resistance and contribute to maintaining the stemness of CSCs including the CD44^+^/CD24^−^ phenotype and the mammosphere forming ability^13^, while the CD44^+^/CD29^+^ cells in squamous cell carcinoma showed properties of CSCs and EMT^12^. Besides, CD44 and ALDH1 are considered as specific markers of CSCs in colon cancer^25^, gastric cancer^26^, and squamous cell carcinoma^27^, the expression of ALDH1 in breast cancer may contribute to a more aggressive phenotype^28^. CD44^+^ALDH1^+^ keratinocytes in normal skin also exhibited properties of stem cells, including self-renewal, enhanced colony formation, long-term epidermal regeneration, and multipotency^29^. In our study, CD44 and ALDH1 were found to be up-regulated and together with the increased sphere formation ability in keloid keratinocytes transfected with mir-21-5p mimic, which cells were undergoing EMT as well. The results indicated that mir-21-5p might have crucial effects on the invasion and the recurrence of keloids by inducing the EMT phenotype and gaining the stem-like cells signatures in keloid keratinocytes. This link between EMT and cells stemness suggested EMT could be a resource for the generation of stem-like keratinocytes in keloid.

An increasing number of studies demonstrated that miR-21 could regulate the CSC phenotype and EMT that are related to invasion in various cancers by targeting PTEN in AKT pathways. For example, knocking down of miR-21 reversed the phenotype of EMT and CSC, and impaired the sphere formation in breast cancer or pediatric cancer via AKT and ERK1/2 pathways by targeting PTEN^10^,^30^. The overexpression of miR-21 increased Akt phosphorylation and decreased the sensitivity of gastric cancer cells to trastuzumab by down-regulation of PTEN^31^ while the inhibition of miR-21 could increase the radiosensitivity in esophageal cancer cells^32^. PTEN has essential roles in the activation and lineage fate determination of hematopoietic stem cells^33^. However, it is unclear whether the role of miR-21 on EMT and stem-like cells phenotype in keloid epithelial cells is to affect PTEN, AKT, or ERK1/2 pathways. We found that miR-21-5p could target and regulate the *PTEN* gene with changes in AKT phosphorylation to further influence the EMT phenotype and cells stemness of keloid keratinocytes. In addition, in the PTEN knockdown only group, there was a remarkable increasing for the expressions of vimentin and ALDH1 in keloid keratinocytes, which confirmed the role of PTEN in the invasive phenotype of keloids. These data indicated that mir-21-5p might play a key role in the invasion and recurrence of keloids through the AKT signaling pathway by PTEN regulation.

Another interesting observation in our study is the responsiveness of normal skin keratinocytes to miR-21-5p. Although EMT induction was not induced in miR-21-5p transfected normal skin keratinocytes, the expressions of vimentin and E-cadherin in mimic or inhibitor transfected normal skin keratinocytes showed the similar change tendency, which indicated that there might be other moleculars targeted by miR-21-5p and to prevent the decreasing of E-cadherin in normal skin keratinocytes. Therefore, normal skin keratinocytes wouldn’t gain abnormal ability of the migration and invasion. Keloid keratinocytes not only exhibited an increased miR-21-5p level, but also were uniquely able to respond to changes in miR-21-5p levels in our study. Indeed, keloid fibroblasts were also found to be more sensitive to Wnt3a treatment compared with normal fibroblasts^34^. The different responsiveness to the environmental stimulation could be a real difference between keloid cells and normal skin cells, and the underlying mechanism might give the new light on keloid pathogenesis. However, it should be noted that keratinocytes from varied locations, including back, shoulder, earlobe and abdomen, were selected for the transfection experiments in our study, although the expression tendencies of genes we analyzed were consistent in both normal and keloid groups, the impact of different body location on the variability in gene expression among keratinocytes might be considered as well.

In conclusion, our data showed the expression of miR-21-5p was up-regulated in keloid epidermis which might account for the invasion and recurrence of keloids due to the enhanced EMT phenotype and the stem-like cells characteristics of keloid keratinocytes. In addition, we demonstrated that the regulation of miR-21-5p on keloid keratinocytes involved the AKT signaling pathway by targeting the *PTEN* gene. Our study revealed a molecular mechanism for the formation of keloids, suggesting that mir-21-5p and its associated regulators of EMT, stem-like cells, and AKT signaling pathway could be novel therapeutic targets for keloids.

## Methods

All experimental procedures were carried out following the manufacturer’s instructions unless otherwise stated.

### Human tissue specimens

Keloid samples were derived from 8 unrelated Chinese patients who underwent surgical excision and had never been treated for keloids. Normal skin samples were obtained from 8 healthy donors during plastic surgery procedures. The characteristics of the samples were presented in Supplementary Table S1. All procedures in this study were approved by the Ethics Committee of the Plastic Surgery Hospital and all patients provided written informed consent. All experiments were performed in accordance with relevant guidelines and regulations.

### MicroRNA microarray analysis for skin epidermis

Strips of keloid and normal skin tissues were incubated in 2.5 mg/ml dispase solution (Sigma-Aldrich, St Louis, MO) at 4 °C for 16 hours, and the epidermis and dermis were separated. Total RNA of epidermis were extracted using mirVana™ miRNA Isolation Kit (Ambion, Austin, TX) and RNA integration was checked using the Agilent 2100 Bioanalyzer (Agilent Technologies, Santa Clara, CA). Samples from 3 keloids and 3 normal skin with the RNA integrity number (RIN) ≥ 6.0 and 28S/18S ratio ≥0.7 were detected using Agilent’s Human miRNA Microarray Release 18.0 (containing 1887 human microRNAs, Agilent Technologies) at Shanghai Biochip Co., Ltd., China. The slides were scanned by Agilent Microarray Scanner with Feature Extraction Software 10.7, and raw data were normalized by Quantile algorithm, Gene Spring Software 11.0 (all by Agilent Technologies). Differentially expressed microRNAs were identified by significance analysis of microarrays (SAM, http://www-stat.stanford.edu/tibs/SAM/index.html). MiRNA expression was considered down- or up-regulated if the fold change in expression level (Keloid/Normal skin) was <−2 or >2 and a false discovery rate (FDR) corrected p-value was <0.05.

### Quantitative RT PCR of miRNAs

To validate the data from miRNA microarray, 3 up-regulated miRNAs were detected by SYBR Green quantitative reverse transcription PCR (qRT-PCR) method in epidermis from 8 keloids and 8 normal skin samples. RT was performed using iScriptcDNA synthesis kit(BIO-RAD, Hercules, CA) and q-PCR was carried out using ABI Power SYBR Green PCR Master Mix (ABI, Foster, CA) in 7900 HT Sequence Detection System (ABI). The comparative threshold cycle (CT) method was used to measure PCR amplification fold differences. The expression of each miRNA was normalized by the expression of the U6 miRNA. All primers were synthesized at Invitrogen, Beijing, China. Primers sequences (5′-3′) were: hsa-mir-146a-5p: TGAGAACTGAATTCCATGGGTT; hsa-mir-197-5p: GTAGAGAGGGCAGTGGGAAAA; hsa-mir-21-5p: TAGCTTATCAGACTGATGTTGA; and U6: TTCGTGAAGCGTTCCATATTTT.

### Culture of keratinocytes from keloid and normal skin and the transfection with miR-21-5p mimic and inhibitor

The keloid and normal skin epithelial sheet were incubated in 0.25% trypsin with 0.02 M ethylene diaminetetraacetic acid (EDTA) at 37 °C for 15 min. Cell suspensions were filtered through 100 μm cell strainers and centrifuged at 1000 rpm for 5 min. Keratinocytes were then resuspended and cultured in keratinocyte-SFM Basal Medium (KM) supplemented with bovine pituitary extract (BPE, 30 μg/ml), recombinant epidermal growth factor (rEGF, 0.2 ng/ml), 100 U/ml penicillin, and 100 mg/ml streptomycin (Invitrogen, Grand Island, NY) at 37 °C in 5% CO_2_. Keratinocytes from 3 keloid and 3 normal skin samples (see Supplementary Table S1) were used in our transfection experiments. Hsa-miR-21-5p mimic (2.5 μl, 25 pmole) and inhibitor (7.5 μl, 75 pmole) and their corresponding mirVana™ miRNA mimic and inhibitor negative control (Ambion) were transfected into the cells using Lipofectamine RNAiMAX in OptiMEM I (Invitrogen). Total RNAs and proteins were extracted simultaneously using mirVana™ PARIS™ Kit (Ambion) after the cells were transfected with the miR-21-5p mimic for 72 hours or with the inhibitor for 48 hours. Expression of miR-21-5p was detected to confirm the transfection. Three replicates were done in each experiment.

### MTS and apoptosis assay

After keloid and normal skin keratinocytes were transfected with miR-21-5p mimic (2.5 μl, 25 pmole) or inhibitor (7.5 μl, 75 pmole) and the corresponding negative controls for 72 or 48 hours, the MTS assay was performed using CellTiter 96® AQueous One Solution Cell Proliferation Assay (Promega, Madison, WI), and the absorbance (OD 490 nm) was used to evaluate the viable cells. The apoptotic cells were detected using Annexin VPE apoptosis detection kit (eBioscience, San Diego, CA).

### Migration and invasion assay

For migration assay, the cells were transfected with hsa-miR-21-5p mimic for 48 hours or with hsa-miR-21-5p inhibitor for 24 hours, then 5 × 10^4^ cells were seeded on a 8 μm pore size insert of Transwell (Millipore, Darmstadt, Germany), or the insert coated with Matrigel (1:2) (BD, San Jose, NJ) for the invasion assay. After 24-hour incubation in KM with BPE and rEGF, cells that adhered to the upper surface of the insert were removed, and those on the under surface were stained with 0.2% crystal violet. After being dissolved in isopropanol, the optical density at 595 nm (OD_595nm_) was detected.

### Immunofluorescence double staining

After keloid and normal skin keratinocytes were transfected with miR-21-5p mimic (2.5 μl, 25 pmole) or inhibitor (7.5 μl, 75 pmole) and the corresponding negative controls for 72 or 48 hours, cells were fixed in 4% paraformaldehyde for 30 min at room temperature. Then the cells were permeabilized in 0.1% Triton X-100 for 5 min and blocked for 30 min with 1% bovine albumin. The cells were then incubated overnight at 4 °C with primary antibodies targeting CD44 (rabbit polyclonal, 1:25, Proteintech Group, Chicago, IL) and vimentin (mouse monoclonal, 1:100, Santa Cruz Biotechnology) and developed with Alexa Fluor 488-conjugated donkey anti-rabbit immunoglobulin G (1:300) (Molecular Probes/Invitrogen) and Cy3-conjugated sheep anti-mouse antibodies (1:200) (Sigma-Aldrich, St. Louis, MO, USA). The slides were mounted with a solution containing DAPI (Molecular Probes/Invitrogen) and imaged with a Leica DM3000 microscope (Leica Microsystems Gmbh, Wetzlar, Germany).

### Sphere-forming assay

After the cells were transfected with miR-21-5p mimic for 48 hours or miR-21-5p inhibitor for 24 hours, 2.5 × 10^5^ cells/well were cultured in DMEM/F12 (1:1) with B27 (Invitrogen), 40 ng/ml FGE-2, 20 ng/ml EGF (Peprotech, Rocky Hill, NJ) in a 6-well ultra-low adherent plate (Corning, Corning, NY) for 7 days. Spheres >50 μm in diameter were counted, digested with trypsin, and resuspended. The total number of cells in the single-cell suspension was counted; the average number of cells in each sphere was calculated using the total cell number divided by the number of spheres.

### RNA interference of PTEN and antagonism of miR-21-5p

The selected RNA duplex (siPTEN) specific for the sequence of PTEN mRNA and the scramble control siRNA sequence (SsiPTEN) were transfected into cells with Transfection Reagent in Transfection Medium (Santa Cruz Biotechnology, Santa Cruz, CA) at a final concentration of 80 nM (8 μl). After 24 hours, the miR-21-5p inhibitor (7.5 μl, 75 pmole) or the negative control (Ambion) were transfected into the cells using LipofectamineRNAiMAX in OptiMEM I (Invitrogen). Proteins were extracted using mirVana™ PARIS™ Kit (Ambion) after the cells were transfected with the miR-21-5p inhibitor for 48 hours.

### Quantitative RT PCR of mRNA

Quantitative RT PCR (qRT-PCR) was performed using FastStart Universal SYBR Green Master Kit (ROX, Roche Applied Science, Mannheim, Germany) and a LightCycler 480II RealTime PCR System (Roche). The CT method was used to measure PCR amplification fold differences. Glyceraldehyde-3-phosphate dehydrogenase (GAPDH) was used to normalize the expression level of each gene. All primers were synthesized at Beijing Invitrogen, China. Primer sequences were: *E-cadherin* (sense) 5′-TTCCTCCCAATACATCTCCC-3′ and (antisense) 5′-TTGATTTTGTAGTCACCCACC-3′; *vimentin* (sense) 5′-CTCTTCCAAACTTTTCCTCCC -3′ and (antisense) 5′-AGTTTCGTTGATAACCTGTCC-3′; *Snail 1* (sense) 5′-TAGCGAGTGGTTCTTCTGC-3′ and (antisense) 5′-GCTGGAAGGTAAACTCTGG-3′; *Snail 2* (sense) 5′-CTCCAAAAAGCCAAACTACAG-3′ and (antisense) 5′-GAGAGAGGCCATTGGGTAG-3′; and *GAPDH* (sense) 5′-GAAGGTGAAGGTCGGAGT-3′ and (antisense) 5′- GAGATGGTGATGGGATTTC-3′.

### Western blot analysis

The specific primary antibodies against E-cadherin, p-ERK (rabbit polyclonal, 1:100, Santa Cruz Biotechnology), and PTEN, vimentin (mouse monoclonal, 1:100, Santa Cruz Biotechnology), CD44 (rabbit polyclonal, 1:2000, Proteintech Group, Chicago, IL), ALDH1 (rabbit polyclonal, 1:2000, GeneTex, Irvine, CA), ERK (rabbit polyclonal, 1:200, GeneTex), p-Akt and Akt (rabbit polyclonal, 1:1000, Cell Signaling Technology, Beverly, MA), and horseradish peroxidase-conjugated anti-mouse or anti-rabbit secondary antibody (1:4000, Santa Cruz Biotechnology) were used in western blot analysis. Protein bands were visualized using a chemiluminescent kit (Super-signal West Pico kit, Pierce, Rockford, IL) and Kodak X-ray films. GAPDH was used (mouse monoclonal, 1:1000, Santa Cruz Biotechnology) as the standard for protein quantitative analysis.

### Statistical analysis

Statistical significance was determined using t-test and one-way analysis of variance (ANOVA) by GraphPad Prism 5.0 software (GraphPad software, Inc., La Jolla, Calif). Mean differences were considered statistically significant if *P* < 0.05. Data were shown as mean ± SEM (standard errors of the mean).

## Additional Information

**How to cite this article**: Yan, L. *et al.* MiR-21-5p Links Epithelial-Mesenchymal Transition Phenotype with Stem-Like Cell Signatures via AKT Signaling in Keloid Keratinocytes. *Sci. Rep.* **6**, 28281; doi: 10.1038/srep28281 (2016).

## Supplementary Material

Supplementary Information

## Figures and Tables

**Table 1:** MicroRNAs differential expressions of epidermis in keloids.

**MicroRNA**	**Fold change (keloid epidermis/normal skin epidermis)**	**p values**
*hsa-miR-513a-5p*	170.6	0.0019
*hsa-miR-374b-5p*	152.6	0.015
*hsa-miR-21-5p*	3.11	0.02
*hsa-miR-197-5p*	2.21	0.013
*hsa-miR-146a-5p*	2.17	0.048
*hsa-miR-3653*	96.4	0.025
*hsa-miR-4649-3p*	60.7	0.011
*hsa-miR-3156-5p*	2.11	0.042
