# Supplementary material for: MiR-21-5p Links Epithelial-Mesenchymal Transition Phenotype with Stem-Like Cell Signatures via AKT Signaling in Keloid Keratinocytes
Source: Sci Rep. 2016 Sep 6;6:28281. doi: 10.1038/srep28281 (PMC5011940; doi:10.1038/srep28281)
Supplement: Supplementary Information [file srep28281-s1.doc]

**Supplementary Information**

**Title: MiR-21-5p Links Epithelial-Mesenchymal Transition Phenotype with Stem-Like Cell Signatures via AKT Signaling in Keloid Keratinocytes**

**Authors:** Li Yan^1^, Rui Cao^1^, YuanBo Liu^2^, LianZhao Wang^3^, Bo Pan^4^, XiaoYan Lv^1^, Hu Jiao^1^, Qiang Zhuang^1^, XueJian Sun^1^, Ran Xiao^1^*

**Supplementary Table S1. Samples Data**

| **Case** | **Age**  **(years)** | **Sex** | **Site** | **Cause** | **MiRNA microarray**  **analysis of epidermis** | **qRT-PCR**  **detection of epidermis** | **MiRNA-21-5p transfection**  **of keratinocytes** |
| --- | --- | --- | --- | --- | --- | --- | --- |
| Keloid 1 | 26 | Male | Jaw | Trauma | √ | √ |  |
| Keloid 2 | 24 | Female | Back | Trauma | √ | √ | √ |
| Keloid 3 | 19 | Female | Shoulder | Trauma | √ | √ | √ |
| Keloid 4 | 30 | Female | Earlobe | Piercing |  | √ |  |
| Keloid 5 | 23 | Male | Earlobe | Trauma |  | √ | √ |
| Keloid 6 | 19 | Female | Earlobe | Piercing |  | √ |  |
| Keloid 7 | 25 | Female | Earlobe | Piercing |  | √ |  |
| Keloid 8 | 21 | Female | Earlobe | Piercing |  | √ |  |
| Normal skin 1 | 21 | Female | Back | Burn |  | √ | √ |
| Normal skin 2 | 25 | Female | Abdomen | Trauma | √ | √ | √ |
| Normal skin 3 | 48 | Male | Abdomen | Trauma | √ | √ |  |
| Normal skin 4 | 20 | Male | Abdomen | Trauma | √ | √ | √ |
| Normal skin 5 | 8 | Male | Abdomen | Burn |  | √ |  |
| Normal skin 6 | 21 | Male | Abdomen | Burn |  | √ |  |
| Normal skin 7 | 20 | Male | Abdomen | Burn |  | √ |  |
| Normal skin 8 | 30 | Male | Abdomen | Trauma |  | √ |  |

**Note: “√” mean the epidermis or keratinocytes from samples were used in the experiments**
